# Vaccination Coverage for Selected Vaccines and Exemption Rates Among Children in Kindergarten — United States, 2017–18 School Year

**DOI:** 10.15585/mmwr.mm6740a3

**Published:** 2018-10-12

**Authors:** Jenelle L. Mellerson, Choppell B. Maxwell, Cynthia L. Knighton, Jennifer L. Kriss, Ranee Seither, Carla L. Black

**Affiliations:** ^1^Certified Technical Experts Inc., Montgomery, Alabama; ^2^National Center for Immunization and Respiratory Disease, Immunization Services Division, CDC.

State and local school vaccination requirements exist to ensure that students are protected from vaccine-preventable diseases (*1*). This report summarizes vaccination coverage and exemption estimates collected by state and local immunization programs* for children in kindergarten (kindergartners) in 49 states and the District of Columbia (DC) and kindergartners provisionally enrolled (attending school without complete vaccination or exemption while completing a catch-up vaccination schedule) or in a grace period (a set interval during which a student may be enrolled and attend school without proof of complete vaccination or exemption) for 28 states. Median vaccination coverage^†^ was 95.1% for the state-required number of doses of diphtheria and tetanus toxoids, and acellular pertussis vaccine (DTaP); 94.3% for 2 doses of measles, mumps, and rubella vaccine (MMR); and 93.8% for 2 doses of varicella vaccine. The median percentage of kindergartners with an exemption from at least one vaccine^§^ was 2.2%, and the median percentage provisionally enrolled or attending school during a grace period was 1.8%. Vaccination coverage among kindergartners remained high; however, schools can improve coverage by following up with students who are provisionally enrolled, in a grace period, or lacking complete documentation of required vaccinations.

Federally funded immunization programs collaborate with departments of education, school nurses, and other school personnel to assess vaccination coverage and exemption status of children enrolled in public and private kindergartens.^¶^ In accordance with state and local school entry requirements, parents and guardians submit children’s vaccination records or exemption forms to schools, or schools obtain records from state immunization information systems. During the 2017–18 school year, 49 states and DC reported coverage for all state-required vaccines and exemption data among public school kindergartners; 48 states and DC reported on private school kindergartners.** Median vaccination coverage for the state-required number of doses of DTaP, 2 doses of MMR, and 2 doses of varicella vaccine are reported. Coverage with hepatitis B and poliovirus vaccines, which are required in most states but not included in this report, are presented on SchoolVaxView (*2*). Twenty-eight states reported data on kindergartners who, at the time of assessment, attended school under a grace period or provisional enrollment. Immunization programs in U.S. territories also receive public funding for immunization and report vaccination coverage and exemptions to CDC; however, national medians and summary measures reported here include only the U.S. states and DC.

Vaccination coverage and exemption estimates were adjusted according to survey type and response rates.^††^ During the 2017–18 school year, vaccination coverage data were reported for approximately 3,988,127 kindergartners, exemption data for approximately 3,634,631, and grace period and provisional enrollment data for approximately 2,825,691.^§§^ Potentially achievable coverage for MMR was calculated for each state as the percentage of students vaccinated with 2 doses of MMR plus the percentage without 2 doses of MMR and no documented vaccination exemption. Nonexempt students included those provisionally enrolled, in a grace period, or otherwise without documentation of vaccination.

During the 2017–18 school year, vaccination assessments varied by immunization program because of differences in states’ required vaccines and doses, vaccines assessed, assessment methods, and data reported. Among the 49 states and DC reporting kindergarten vaccination data, 36 used a census; nine used a sample; three used a voluntary school response; and two used a mix of sampling methods.^¶¶^ All states used the same methods to collect both vaccination coverage and exemption data except Alaska, Kansas, Virginia, and Wisconsin, where a sample was used for vaccination coverage data and a census for exemption data. Kindergartners were considered up to date and included in the coverage estimate for a given vaccine if they received all doses required for school entry,*** except in seven states^†††^ that considered kindergartners up to date only if they received all doses of all vaccines required for school entry. Reporting of varicella vaccination status among kindergartners with a history of varicella disease varied within and among states; some were reported as vaccinated against varicella and others as medically exempt.

Among the 49 states and DC included in this analysis, median 2-dose MMR coverage was 94.3% (range = 81.3% [DC] to ≥99.4% [Mississippi]), 23 states reported coverage ≥95%, and three states and DC reported coverage <90% (Table 1). Median DTaP coverage was 95.1% (range = 79.7% [DC] to ≥99.4% [Mississippi]), 25 states reported coverage ≥95%, and three states and DC reported coverage <90%. Among the 41 states and DC that required and reported 2 doses of varicella vaccine, median coverage was 93.8% (range = 80.5% [DC] to ≥99.4% [Mississippi]), 17 states reported coverage ≥95%, and four states and DC reported coverage <90%.

**TABLE 1:** Estimated vaccination coverage* for MMR, DTaP, and varicella vaccines among children enrolled in kindergarten, by vaccine and immunization program — United States and territories, 2017–18 school year

Immunization program	Kindergarten population^†^	No. (%) surveyed	Type of survey conducted^§^	Local data available online^¶^	MMR**	DTaP^††^	Varicella			
					2 doses (%)	4 or 5 doses (%)	1 dose (%)		2 doses (%)	
**Median^§§^**					**94.3**	**95.1**	**96.2**		**93.8**	
Alabama^¶¶^	57,245	57,245 (100.0)	Census	Yes	≥92.7	≥92.7	≥92.7	NReq		
Alaska***^,†††^	9,692	707 (7.3)	Stratified 2-stage cluster sample	No	91.6	91.1	NA	91.3		
Arizona^¶¶^	81,710	81,710 (100.0)	Census	Yes	93.4	93.5	96.2	NReq		
Arkansas^§§§^	39,630	38,242 (96.5)	Census (public), voluntary response (private)	No	91.9	91.3	NA	91.6		
California^§§§^	574,702	564,121 (98.2)	Census	Yes	96.9	96.4	98.2	NReq		
Colorado^¶¶^	65,718	65,718 (100.0)	Census	Yes	88.7	88.6	NA	87.7		
Connecticut^¶¶^	39,174	39,174 (100.0)	Census	No	96.5	96.5	NA	96.3		
Delaware	10,988	1,053 (9.6)	Stratified 2-stage cluster sample	No	96.7	96.9	NA	96.7		
District of Columbia^¶¶^	8,205	8,205 (100.0)	Census	No	81.3	79.7	NA	80.5		
Florida^¶¶,^***	222,397	222,397 (100.0)	Census	Yes	≥93.7	≥93.7	NA	≥93.7		
Georgia^¶¶^	131,459	131,459 (100.0)	Census	No	≥93.4	≥93.4	NA	≥93.4		
Hawaii	16,325	1,040 (6.4)	Stratified 2-stage cluster sample	No	95.6	95.4	96.2	NReq		
Idaho	22,553	22,458 (99.6)	Census	Yes	89.5	89.3	NA	88.6		
Illinois^¶¶^	144,858	144,858 (100.0)	Census	Yes	95.2	95.3	NA	94.8		
Indiana	84,296	70,857 (84.1)	Voluntary response	Yes	90.4	94.3	NA	90.2		
Iowa^¶¶^	39,632	39,632 (100.0)	Census	Yes	≥93.0	≥93.0	NA	≥93.0		
Kansas***^,†††,§§§^	38,484	8,728 (22.7)	Stratified 2-stage cluster sample	Yes	89.1	89.5	NA	88.3		
Kentucky***^,§§§^	55,152	50,538 (91.6)	Census	Yes	92.6	93.7	NA	91.7		
Louisiana^¶¶^	58,277	58,277 (100.0)	Census	Yes	96.1	97.7	NA	95.6		
Maine	13,255	12,527 (94.5)	Census	Yes	94.3	95.3	96.5	NReq		
Maryland^§§§^	68,528	67,747 (98.9)	Census	No	98.6	99.0	NA	98.6		
Massachusetts^¶¶,§§§^	63,377	63,377 (100.0)	Census	Yes	96.3	96.4	NA	96.0		
Michigan^¶¶^	119,028	119,028 (100.0)	Census	Yes	95.0	95.3	NA	94.7		
Minnesota***	69,807	67,372 (96.5)	Census	Yes	92.5	92.8	NA	92.2		
Mississippi^¶¶^	39,284	39,284 (100.0)	Census	Yes	≥99.4	≥99.4	NA	≥99.4		
Missouri^¶¶^	73,113	73,113 (100.0)	Census	No	95.2	95.3	NA	95.0		
Montana^¶¶^	12,188	12,188 (100.0)	Census	No	93.2	92.6	NA	91.6		
Nebraska^§§§^	26,313	25,796 (98.0)	Census	No	96.2	96.7	NA	95.5		
Nevada	37,178	1,769 (4.8)	Stratified 2-stage cluster sample	No	93.0	92.6	NA	92.6		
New Hampshire	12,165	11,939 (98.1)	Census	No	≥92.4	≥92.4	NA	≥92.4		
New Jersey^¶¶^	107,630	107,630 (100.0)	Census	Yes	≥96.1	≥96.1	≥96.1	NReq		
New Mexico	26,896	1,256 (4.7)	Stratified 2-stage cluster sample	No	94.8	94.9	NA	94.5		
New York (including New York City)^¶¶^	226,456	226,456 (100.0)	Census	Yes	97.2	96.9	NA	96.9		
New York City^¶¶^	100,466	100,466 (100.0)	Census	No	97.8	97.3	NA	97.4		
North Carolina***^,§§§^	127,197	120,827 (95.0)	Census	No	97.0	96.8	NA	96.8		
North Dakota	10,365	10,293 (99.3)	Census	Yes	94.2	94.1	NA	93.9		
Ohio	138,753	132,763 (95.7)	Census	No	92.1	92.1	NA	91.5		
Oklahoma***	53,898	48,481 (89.9)	Census (public), voluntary response (private)	No	92.6	93.9	96.8	NReq		
Oregon^¶¶,§§§^	45,818	45,818 (100.0)	Census	Yes	93.2	92.4	94.4	NReq		
Pennsylvania	141,571	123,377 (87.1)	Voluntary response	Yes	96.7	97.0	NA	97.0		
Rhode Island^¶¶,^***^,§§§^	11,025	11,025 (100.0)	Census	Yes	96.4	96.2	NA	96.0		
South Carolina	58,458	16,174 (27.7)	Stratified 1-stage cluster sample	No	96.3	96.6	NA	96.1		
South Dakota	12,125	12,112 (99.9)	Census	Yes	96.6	95.9	NA	95.8		
Tennessee^¶¶,^***	78,743	78,743 (100.0)	Census	Yes	96.9	96.7	NA	96.8		
Texas (including Houston)***^,§§§^	387,981	378,008 (97.4)	Census	Yes	96.9	96.8	NA	96.4		
Houston***^,§§§^	43,340	38,343 (88.5)	Voluntary response (public), Census (private)	No	95.1	95.2	NA	94.7		
Utah^¶¶^	48,827	48,827 (100.0)	Census	Yes	93.4	93.2	NA	93.7		
Vermont^¶¶^	6,255	6,255 (100.0)	Census	Yes	94.1	94.0	NA	93.2		
Virginia^†††^	100,581	4,224 (4.2)	Stratified 2-stage cluster sample	Yes	95.5	98.2	NA	93.3		
Washington***	85,118	79,977 (94.0)	Census	Yes	90.6	90.7	NA	89.4		
West Virginia****	19,519	15,120 (77.5)	Voluntary response	Yes	98.4	98.0	NA	98.1		
Wisconsin***^,†††,§§§^	66,178	1,223 (1.8)	Stratified 2-stage cluster sample	No	91.8	96.5	NA	91.2		
Wyoming	NA	NA	Not conducted	No	NA	NA	NA	NA		
**Territories and associated states**									
American Samoa^¶¶,^****	758	758 (100.0)	Census	No	90.9	81.8	NReq	NReq		
Federated States of Micronesia^¶¶^	1,886	1,886 (100.0)	Census	No	94.0	75.8	NReq	NReq		
Guam	2,625	700 (26.7)	Stratified 2-stage cluster sample	No	85.0	92.0	NReq	NReq		
Marshall Islands^¶¶^	1,086	1,086 (100.0)	Census	No	96.6	67.7	NReq	NReq		
Northern Mariana Islands^¶¶^	876	876 (100.0)	Census	No	92.8	75.6	NA	92.6		
Palau^¶¶,¶¶¶^	313	313 (100.0)	Census	No	100.0	100.0	NReq	NReq		
Puerto Rico^††††^	NA	NA	Not conducted	No	NA	NA	NA	NA		
U.S. Virgin Islands^††††^	NA	NA	Not conducted	No	NA	NA	NA	NA		

**Abbreviations:** DTaP/DT = diphtheria and tetanus toxoids (DT) and acellular pertussis vaccine; MMR = measles, mumps, and rubella vaccine; NA = not available; NReq = not required for school entry.

* Estimates are adjusted for nonresponse and weighted for sampling where appropriate. Estimates based on a completed vaccine series (i.e., not vaccine-specific) use the “≥” symbol. Coverage might include history of disease or laboratory evidence of immunity.

^†^ The kindergarten population is an approximation provided by each program.

^§^ Sample designs varied by state or area: census = program attempted to include all schools (public and private) and all children within schools in the assessment and had a student response rate of ≥90%; 1-stage or 2-stage cluster sample = schools were randomly selected, and all children in the selected schools were assessed (1-stage), or a random sample of children within the schools was selected (2-stage); voluntary response = a census with a student response rate of <90% (does not imply that participation was optional).

^¶^ Some programs publish kindergarten vaccination data online that are more detailed than the state-level estimates in this table. Examples of more detailed data include county, parish, school district, and school-level estimates.

** Most states require 2 doses of MMR; Alaska, New Jersey, and Oregon require 2 doses of measles, 1 dose of mumps, and 1 dose of rubella vaccines. Georgia, New York, New York City, North Carolina, and Virginia require 2 doses of measles and mumps and 1 dose of rubella vaccines. Iowa requires 2 doses of measles and 2 doses of rubella vaccines.

^††^ Pertussis vaccination coverage might include some diphtheria, tetanus toxoids, and pertussis vaccine (DTP) vaccinations if administered in another country or by a vaccination provider who continued to use DTP after 2000. Most states require 5 doses of DTaP for school entry, or 4 doses if the fourth dose was received on or after the fourth birthday; Illinois, Maryland, Virginia, and Wisconsin require 4 doses; Nebraska requires 3 doses. The reported coverage estimates represent the percentage of kindergartners with the state-required number of DTaP doses, except for Kentucky, which requires ≥5 but reports ≥4 doses of DTaP.

^§§^ Medians calculated from data from 49 states and the District of Columbia (i.e., does not include Wyoming, Houston, New York City, American Samoa, Federated States of Micronesia, Guam, Marshall Islands, Northern Mariana Islands, Palau, Puerto Rico, or U.S. Virgin Islands). Coverage data were reported for 3,988,127 kindergartners.

^¶¶^ The percentage surveyed likely was <100%, but is reported as 100% based on incomplete information about the actual current enrollment.

*** Did not include some types of schools, such as online schools or those located on military bases or in correctional facilities.

^†††^ Kindergarten vaccination coverage data were collected from a sample, and exemption data were collected from a census of kindergartners.

^§§§^ Counted some or all vaccine doses received regardless of Advisory Committee on Immunization Practices recommended age and time interval; vaccination coverage rates reported might be higher than those for valid doses.

^¶¶¶^ For Palau, estimates represent coverage among children in first grade.

**** Reported public school data only.

^††††^ Puerto Rico and U.S. Virgin Islands did not report data for the 2017–18 school year because of widespread logistical issues caused by Hurricane Maria.

The median percentage of kindergartners with an exemption from one or more required vaccines (not limited to MMR, DTaP, and varicella vaccines) was 2.2% (range = 0.1% [Mississippi] to 7.6% [Oregon]), compared with 2.0% during the 2016–17 school year (Table 2). The median percentage of medical exemptions was 0.2% (range = <0.1% [Hawaii] to 0.8% [Alaska]); the median percentage of nonmedical exemptions was 2.0% (range = <0.1% [California] to 7.5% [Oregon]). Among the 29 states and DC with an increase in exemptions in 2017–18, vaccination coverage was ≥95% in 15 states for MMR, 16 states for DTaP, and 11 states for 2 doses of varicella.

**TABLE 2:** Estimated number and percentage* of children enrolled in kindergarten with reported type of exemption from vaccination, and grace period/provisional enrollment, by immunization program^†^ — United States and territories, 2017–18 school year

Immunization program	Medical exemptions, no. (%)	Nonmedical exemptions			Any exemption				Grace period or provisional enrollment^§^ no. (%)
		Religious no.	Philosophical no.	Total no. (%)	2017–18, no.	2017–18 %	2016–17 %	Percentage point difference (2016–17 to 2017–18)	
**Median^¶^**	**(0.2)**	**—**	**—**	**(2.0)**	**—**	**2.2**	**2.0**	**0.2**	**(1.8)**
Alabama	59 (0.1)	460	—**	460 (0.8)	519	0.9	0.7	0.2	None
Alaska	75 (0.8)	549	—**	549 (6.1)	624	7.0	6.8	0.2	NR
Arizona	400 (0.5)	—^††^	4,336	4,336 (5.3)	4,736	5.8	5.1	0.7	NR
Arkansas	14 (0.1)	213	428	641 (1.6)	655	1.7	1.4	0.3	3,379 (8.5)
California	4,190 (0.7)	—^§§^	—^§§^	5 (<0.1)	4,195	0.7	1.1	-0.4	10,568 (1.8)
Colorado	—^¶¶^	—^¶¶^	—^¶¶^	—^¶¶^	—^¶¶^	—^¶¶^	—^¶¶^	—^¶¶^	NR
Connecticut	126 (0.3)	764	—**	764 (2.0)	890	2.3	2.1	0.2	None
Delaware	3 (0.1)	148	—**	148 (1.3)	151	1.4	1.2	0.2	NR
District of Columbia	58 (0.7)	352	—**	352 (4.3)	410	5.0	1.1	3.9	NR
Florida	1,051 (0.5)	5,394	—**	5,394 (2.4)	6,445	2.9	2.5	0.4	7,349 (3.3)
Georgia	102 (0.1)	3,480	—**	3,480 (2.6)	3,582	2.7	2.8	-0.1	287 (0.2)
Hawaii	4 (<0.1)	514	—**	514 (3.1)	518	3.1	2.8	0.3	37 (0.2)
Idaho	93 (0.4)	—^§§^	—^§§^	1,504 (6.7)	1,597	7.1	6.5	0.6	408 (1.8)
Illinois	—^¶¶^	—^¶¶^	—^¶¶^	—^¶¶^	—^¶¶^	—^¶¶^	—^¶¶^	—^¶¶^	NR
Indiana	156 (0.2)	579	—**	579 (0.7)	735	0.9	1.0	-0.1	NR
Iowa	93 (0.2)	694	—**	694 (1.8)	787	2.0	1.8	0.2	1,356 (3.4)
Kansas	125 (0.3)	544	—**	544 (1.4)	669	1.7	1.8	-0.1	NR
Kentucky	174 (0.3)	623	—**	623 (1.1)	797	1.4	1.1	0.3	NR
Louisiana	61 (0.1)	49	552	601 (1.0)	662	1.1	0.8	0.3	NA
Maine	34 (0.3)	58	608	666 (5.0)	700	5.3	5.0	0.3	186 (1.4)
Maryland	390 (0.6)	614	—**	614 (0.9)	1,005	1.5	1.4	0.1	NR
Massachusetts	166 (0.3)	687	—**	687 (1.1)	853	1.3	1.3	0.0	None
Michigan	251 (0.2)	1,095	3,658	4,753 (4.0)	5,004	4.2	3.7	0.5	719 (0.6)
Minnesota	—^¶¶^	—^¶¶^	—^¶¶^	—^¶¶^	—^¶¶^	—^¶¶^	—^¶¶^	—^¶¶^	NR
Mississippi	38 (0.1)	—^††^	**	—**^,††^	38	0.1	0.1	0.0	165 (0.4)
Missouri	—^¶¶^	—^¶¶^	—^¶¶^	—^¶¶^	—^¶¶^	—^¶¶^	—^¶¶^	—^¶¶^	NR
Montana	48 (0.4)	478	—**	478 (3.9)	526	4.3	3.7	0.6	211 (1.7)
Nebraska	192 (0.7)	394	—**	394 (1.5)	586	2.2	2.0	0.2	463 (1.8)
Nevada	26 (0.1)	1,170	—**	1,170 (3.1)	1,196	3.2	4.4	-1.2	600 (1.6)
New Hampshire	22 (0.2)	334	—**	334 (2.7)	357	2.9	3.2	-0.3	573 (4.7)
New Jersey	171 (0.2)	2,148	—**	2,148 (2.0)	2,319	2.2	1.9	0.3	991 (0.9)
New Mexico	51 (0.2)	394	—**	394 (1.5)	445	1.7	2.3	-0.6	679 (2.5)
New York (incl. New York City)	349 (0.2)	2,199	—**	2,199 (1.0)	2,548	1.1	1.0	0.1	4,170 (1.8)
New York City	85 (0.1)	581	—**	581 (0.6)	666	0.7	0.6	0.1	1,173 (1.2)
North Carolina	284 (0.2)	2,323	—**	2,323 (1.8)	2,607	2.0	1.8	0.2	2,248 (1.8)
North Dakota	31 (0.3)	74	244	318 (3.1)	350	3.4	3.4	0.0	NR
Ohio	336 (0.2)	—^§§^	—^§§^	3,207 (2.3)	3,543	2.6	2.4	0.2	7,367 (5.3)
Oklahoma	91 (0.2)	333	657	991 (1.8)	1,182	2.2	1.9	0.3	NR
Oregon	62 (0.1)	—^§§^	—^§§^	3,427 (7.5)	3,489	7.6	6.7	0.9	NR
Pennsylvania	638 (0.5)	1,600	1,779	3,379 (2.4)	4,017	2.8	2.3	0.5	3,124 (2.2)
Rhode Island	10 (0.1)	110	—**	110 (1.0)	120	1.1	1.2	-0.1	NR
South Carolina	119 (0.2)	1,028	—**	1,028 (1.8)	1,147	2.0	2.0	0.0	328 (0.6)
South Dakota	23 (0.2)	238	—**	238 (2.0)	261	2.2	2.0	0.2	NR
Tennessee	114 (0.1)	1,085	—**	1,085 (1.4)	1,199	1.5	1.3	0.2	1,124 (1.4)
Texas (incl. Houston)	780 (0.2)	—^§§^	—^§§^	7,044 (1.8)	7,825	2.0	1.8	0.2	6,811 (1.8)
Houston	66 (0.2)	—^§§^	—^§§^	459 (1.1)	525	1.2	1.0	0.2	NR
Utah	80 (0.2)	19	2,507	2,526 (5.2)	2,606	5.3	5.1	0.2	1,039 (2.1)
Vermont	13 (0.2)	227	—**	227 (3.6)	240	3.8	3.9	-0.1	321 (5.1)
Virginia	384 (0.4)	1,125	—**	1,125 (1.1)	1,508	1.5	1.2	0.3	NR
Washington	621 (0.7)	202	3,142	3,344 (3.9)	3,966	4.7	4.8	-0.1	1,396 (1.6)
West Virginia***	32 (0.2)	—^††^	—**	—**^,††^	32	0.2	0.3	-0.1	809 (4.1)
Wisconsin	164 (0.2)	291	3,122	3,413 (5.2)	3,577	5.4	5.5	-0.1	1,907 (2.9)
Wyoming	NA	NA	NA	NA	NA	NA	NA	NA	NA
**Territories and associated states**									
American Samoa	0 (0.0)	0	—**	0 (0.0)	0	0	0	0	None
Federated States of Micronesia	0 (0.0)	0	0	0 (0.0)	0	0	0	0.0	NR
Guam	0 (<0.1)	10	—**	10 (0.4)	10	0.4	0.2	0.2	NR
Marshall Islands	0 (0.0)	—^††^	—**	0 (0.0)	0	0	0	0.0	NR
Northern Mariana Islands	0 (0.0)	0	0	0 (0.0)	0	0	0	0.0	NR
Palau^†††^	0 (0.0)	—^§§^	—^§§^	0 (0.0)	0	0	0	0.0	NR
Puerto Rico^§§§^	NA	NA	NA	NA	NA	NA	NA	NA	NA
U.S. Virgin Islands^§§§^	NA	NA	NA	NA	NA	NA	NA	NA	NA

**Abbreviations:** NA = not available (i.e., not collected); None = state does not allow grace period or provisional enrollment; NR = not reported to CDC.

* Estimates are adjusted for nonresponse and weighted for sampling where appropriate.

^†^ Medical exemptions, nonmedical exemptions, and grace period or provisional enrollment status might not be mutually exclusive. Some children might have both medical and nonmedical exemptions, and some enrolled under a grace period or provisional enrollment might be exempt from one or more vaccinations.

^§^ A grace period is a set number of days during which a student can be enrolled and attend school without proof of complete vaccination or exemption. Provisional enrollment allows a student without complete vaccination or exemption to attend school while completing a catch-up vaccination schedule. In states with one or both of these policies, the estimates represent the number of kindergartners within a grace period, provisionally enrolled, or some combination of these categories.

^¶^ Medians calculated from data from 45 states and District of Columbia; states excluded were Colorado, Illinois, Minnesota, Missouri, and Wyoming. Houston, New York City, American Samoa, Federated States of Micronesia, Guam, Marshall Islands, Northern Mariana Islands, Palau, Puerto Rico, and U.S. Virgin Islands also were excluded. Exemption data were reported for 3,634,631 kindergartners. Grace period or provisional enrollment median was calculated from data from 28 states; data were reported for 2,825,691 kindergartners.

** Philosophical exemptions were not allowed.

^††^ Religious exemptions were not allowed.

^§§^ Religious and philosophical exemptions were not reported separately.

^¶¶^ Program did not report the number of children with exemptions, but instead reported the number of exemptions for each vaccine, which could count some children more than once. Lower bounds of the percentage of children with any exemptions estimated using the individual vaccines with the highest number of exemptions are for Colorado, 0.2% with medical exemptions, 0.3% with religious exemptions, 4.2% with philosophical exemptions, and 4.7% with any exemptions; for Illinois, 0.2% with medical exemptions, 1.4% with religious exemptions, and 1.6% with any exemptions; for Minnesota, 0.2% with medical exemptions, 3.4% with nonmedical exemptions, and 3.5% with any exemptions; and for Missouri, 0.2% with medical exemptions, 2.1% with religious exemptions, and 2.3% with any exemptions.

*** Reported public school data only.

^†††^ For Palau, estimates represent exemptions among children in first grade.

^§§§^ Puerto Rico and U.S. Virgin Islands did not report data for the 2017–18 school year because of widespread logistical issues caused by Hurricane Maria.

The median reported percentage of kindergartners attending school during a grace period or provisionally enrolled was 1.8% (range = 0.2% [Georgia and Hawaii] to 8.5% [Arkansas]) (Table 2). In 11 of 28 states reporting for the 2017–18 school year, the percentage of children provisionally enrolled or within a grace period at the time of the assessment exceeded the percentage of children with exemptions from ≥1 vaccines. Among the 26 states and DC with MMR coverage <95%, 20 could potentially achieve ≥95% coverage if all nonexempt students who were provisionally enrolled, in a grace period, or otherwise without evidence of complete vaccination were vaccinated (Figure).

**FIGURE:**
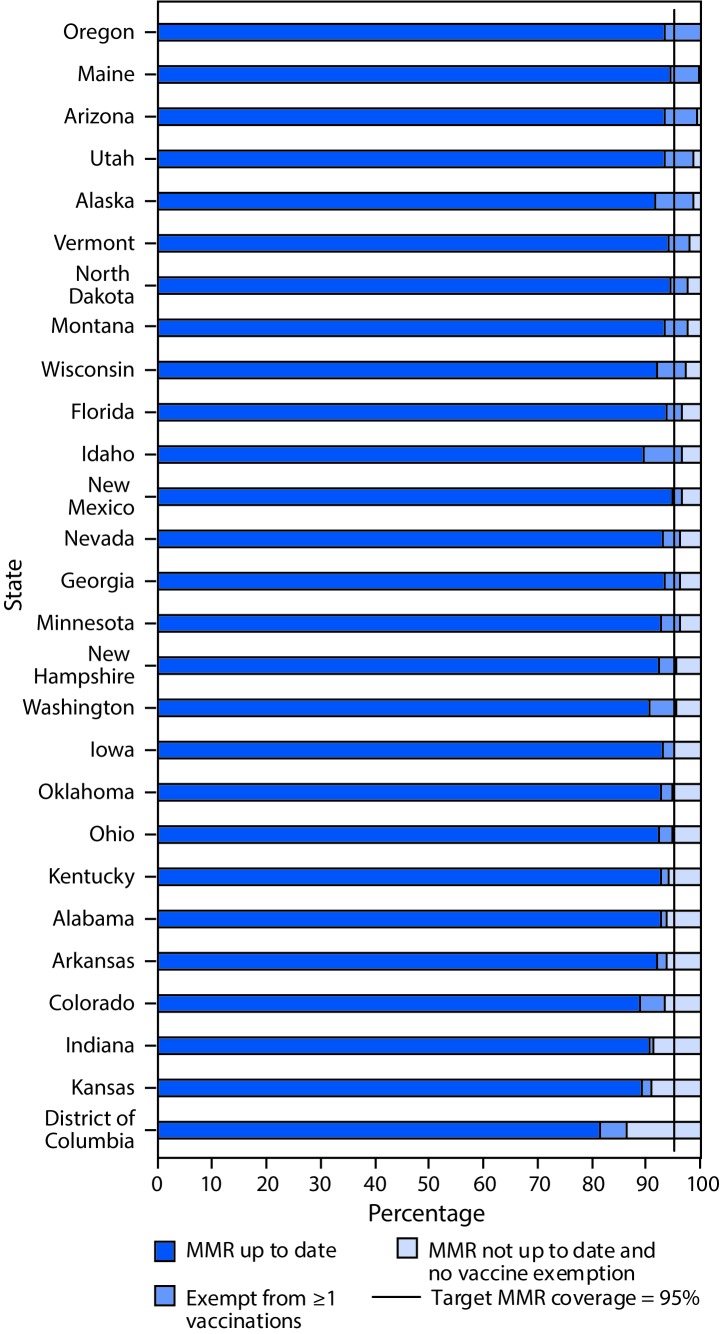
Estimated percentage of kindergartners with documented up-to-date vaccination for measles, mumps, and rubella vaccine (MMR)*; exempt from one or more vaccines^†^^,^^§^; and not up to date with MMR and not exempt^¶^ — selected states and District of Columbia,** 2017–18 school year * Estimates are based on completed vaccine series and are not MMR-specific for Alabama, Florida, Georgia, Iowa, and New Hampshire. Up-to-date coverage reported here is the lower bound of possible MMR coverage. ^†^ Most states report the number of kindergartners with an exemption from one or more vaccines. Estimates reported here might include exemptions from vaccines other than MMR, except in Colorado and Minnesota, where MMR-specific exemptions are reported. ^§^ Coverage estimates are based on a sample of kindergartners, and exemption estimates are based on a census for Alaska, Kansas, and Wisconsin. ^¶^ Includes nonexempt students provisionally enrolled, in a grace period, or otherwise without documentation of complete MMR vaccination. ** Figure includes all states with reported MMR coverage for the 2017–18 school year of <95%, the *Healthy People 2020* target for MMR vaccination coverage among kindergartners. https://www.healthypeople.gov/.

## Discussion

During the 2017–18 school year, median kindergarten vaccination coverage was close to 95% for MMR, DTaP, and varicella vaccine. The number of states with coverage ≥95% increased from 20 to 23 (MMR), 23 to 25 (DTaP), and 15 to 17 (2 varicella vaccine doses) since the 2016–17 school year (*2*,*3*). Coverage increases in selected states might result from modifications to state programs. For example, Pennsylvania reduced its provisional enrollment period from 240 days to 5 days with a medical certificate indicating the scheduling of missing vaccine doses. The Indiana State Department of Health initiated report cards for schools displaying kindergarten vaccination coverage rates and built a bidirectional interface that increased the amount of data in their immunization information system. Kentucky removed the provider signature requirement when printing a certificate of immunization status, allowing school nurses to use the immunization information system certificate to document vaccination history. In Virginia, the number of local health departments participating in back-to-school immunization clinics for children entering school increased, with most local health departments following up with parents about missing vaccinations before the clinics (J Mellerson, CDC, unpublished data, 2018).

Although the overall percentage of children with an exemption was low, this was the third consecutive school year that a slight increase was observed (*2*). Reasons for the increase cannot be determined from the data reported to CDC but could include the ease of the procedure for obtaining exemptions (*4*) or parental vaccine hesitancy (*5*). Reported exemptions do not distinguish between exemptions for one vaccine versus all vaccines. Previous studies indicate that most children with exemptions have received at least some vaccines (*6*–*8*).

Recent data from the National Immunization Survey indicate the percentage of children reaching age 2 years without having received any vaccinations has increased gradually, from 0.9% for children born in 2011 to 1.3% for children born in 2015 (*9*). Two of the 10 states with <90% coverage for ≥1 dose of MMR among children aged 19–35 months in the 2014 National Immunization Survey (*10*) (the approximate cohort of children entering kindergarten in the 2017–18 school year) also had <90% coverage for ≥2 doses of MMR among kindergartners in 2017–18; in eight states, coverage with ≥2 doses of MMR was <95%, indicating that some children who were undervaccinated in early childhood do not catch up before kindergarten entry. This highlights the importance of school entry vaccination requirements to ensure catch-up vaccination of unvaccinated and undervaccinated children.

In 11 of the 28 states reporting 2017–18 grace period or provisional enrollment data, the percentage of kindergartners in these groups at the time of assessment exceeded the percentage with an exemption from one or more vaccines, representing a group of children who could be fully vaccinated with appropriate follow-up. CDC encourages programs to collect and use these data to identify populations of undervaccinated students. Almost all states could achieve ≥95% vaccination coverage if undervaccinated nonexempt children were vaccinated in accordance with local and state vaccination policies.

The findings in this report are subject to at least five limitations. First, comparability is limited because of variation in states’ requirements, data collection methods, and definitions of grace period and provisional enrollment. Second, representativeness might be negatively affected because of data collection methodologies that miss some schools or students or assess vaccination status at different times. Third, actual vaccination coverage, exemption rates, or both might be underestimated or overestimated because of inaccurate or absent documentation. Fourth, median coverage estimates include only 49 of 50 states and DC, median exemption estimates include only 45 states and DC, and the median grace period or provisional enrollment estimate includes only 28 states for the 2017–18 school year. Finally, because most states do not report vaccine-specific exemptions, estimates of potentially achievable MMR coverage are approximations. However, if reported exemptions were for a vaccine or vaccines other than MMR, estimates of potentially achievable MMR coverage would be higher than those presented.

Kindergarten vaccination requirements help ensure that students are fully vaccinated with age-appropriate vaccines upon school entry. Although overall vaccination coverage is high, coverage could be improved in many states. CDC works with immunization programs to collect and report data on school vaccination coverage, exemption rates, and grace period and provisional enrollment each year. Immunization programs can use these data to understand and address undervaccination among kindergartners and to identify schools and communities where focused interventions could improve coverage with required vaccines.

**Summary**

**What is already known about this topic?**

Immunization programs conduct annual kindergarten vaccination assessments to monitor school-entry vaccination coverage for all state-required vaccines.

**What is added by this report?**

Median vaccination coverage was 94.3% for 2 doses of measles, mumps, and rubella vaccine; 95.1% for the state-required number of doses of diphtheria and tetanus toxoids and acellular pertussis vaccine; and 93.8% for 2 doses of varicella vaccine. Although the median exemption rate gradually increased for the third year in a row to 2.2%, most undervaccinated children did not have exemptions.

**What are the implications for public health practice?**

School assessment allows immunization programs to target interventions to schools with undervaccinated kindergartners to increase compliance with state and local vaccination requirements.

## Footnotes

* Federally funded immunization programs are located in the 50 states and DC, five cities, and eight U.S territories and freely associated states (territories). Two cities reported data to CDC, which were included in their state data to calculate medians. Immunization programs in U.S. territories reported vaccination coverage and exemptions to CDC; however, these data were not included in median calculations.

† Median vaccination coverage was determined using estimates for 49 states and DC; Wyoming did not report data because of problems with the quality of data reported by schools. Data from cities were included with their state data. Data from territories were not included in median calculation.

§ Median exemption rate was determined using estimates for 45 states and DC; Wyoming did not report data because of problems with the quality of data reported by schools; Colorado, Illinois, Minnesota, and Missouri were included in the tables and figure but excluded from the median exemption rate because they did not collect information on the number of kindergartners with an exemption. Data from cities were included with their state data. Data from territories were not included in median calculation.

¶ Assessment date varied by state and area. Seven states assess on the first day of school; 18 states assess by December 31; 12 states assess by some other date, ranging from 30 days after admission to March 5; 12 states and DC assess on a rolling basis.

** Six states reported coverage and exemption data for at least some homeschooled kindergartners. California included data for 18 independent study schools and eight virtual schools in public school data and data for homeschools with six or more students in private school data. North Dakota reported some homeschool data separately. Oregon reported some homeschool data separately; children enrolled in public online homeschools were included in the public school data. Pennsylvania included all homeschooled students in their public school data. Utah included some homeschooled students in public and private school data. Vermont included homeschooled students in their public and private school data if the students were enrolled in one or more classes at a school; homeschooled children who were exclusively homeschooled were not subject to vaccination requirements and were not included in these estimates.

†† Most immunization programs that used census or voluntary response provided CDC with data aggregated at the state or local (city or territory) level. Coverage and exemption data based on a census or voluntary response were adjusted for nonresponse using the inverse of the response rate, stratified by school type (public, private, and homeschool, where available). Programs that used complex sample surveys provided CDC with deidentified data aggregated at the school or county level for weighted analysis. Weights were calculated to account for sample design and adjusted for nonresponse for data collected through complex sample design wherever possible.

§§ The kindergarten population is an approximation provided by each immunization program. The totals reported here are the summations of the kindergarten population among programs reporting data for coverage, exemptions, and grace periods or provisional enrollment. Data from cities and territories were not included in these totals.

¶¶ States using a census attempted to collect data from all kindergartners at all schools and succeeded in collecting data for ≥90% of kindergartners. The type of sample employed by the nine states using a sample to collect coverage data varied and included a stratified two-stage cluster sample (eight states) and a stratified one-stage cluster sample (one state). A voluntary response of schools was defined as a census survey with a response rate <90% of the known population of kindergartners. A mix of methods included two or more described sampling methods (a census for one school type and voluntary response for the other).

*** All 49 reporting states and DC required 2 doses of a measles-containing vaccine. Local DTaP requirements varied. Nebraska required 3 doses, four states (Illinois, Maryland, Virginia, and Wisconsin) required 4 doses, and all other states required 5 doses, unless the fourth dose was administered on or after the fourth birthday. The reported coverage estimates represent the percentage of kindergartners with the state-required number of DTaP doses, except for Kentucky, which required 5 doses of DTaP by age 5 years, but reported 4-dose coverage for kindergartners. Nine states required 1 dose of varicella vaccine; 41 states and DC required 2 doses.

††† Alabama, Florida, Georgia, Iowa, Mississippi, New Hampshire, and New Jersey considered kindergartners up to date only if they had received all doses of all vaccines required for school entry.
