# *Erratum:* Vol. 67, No. 40

**DOI:** 10.15585/mmwr.mm6741a9

**Published:** 2018-10-19

In the report “VaccinationCoverageforSelectedVaccinesandExemptionRatesAmongChildreninKindergarten—UnitedStates,2017–18SchoolYear,” on page 1121, an incorrect figure was published. The corrected figure follows.

**FIGURE:**
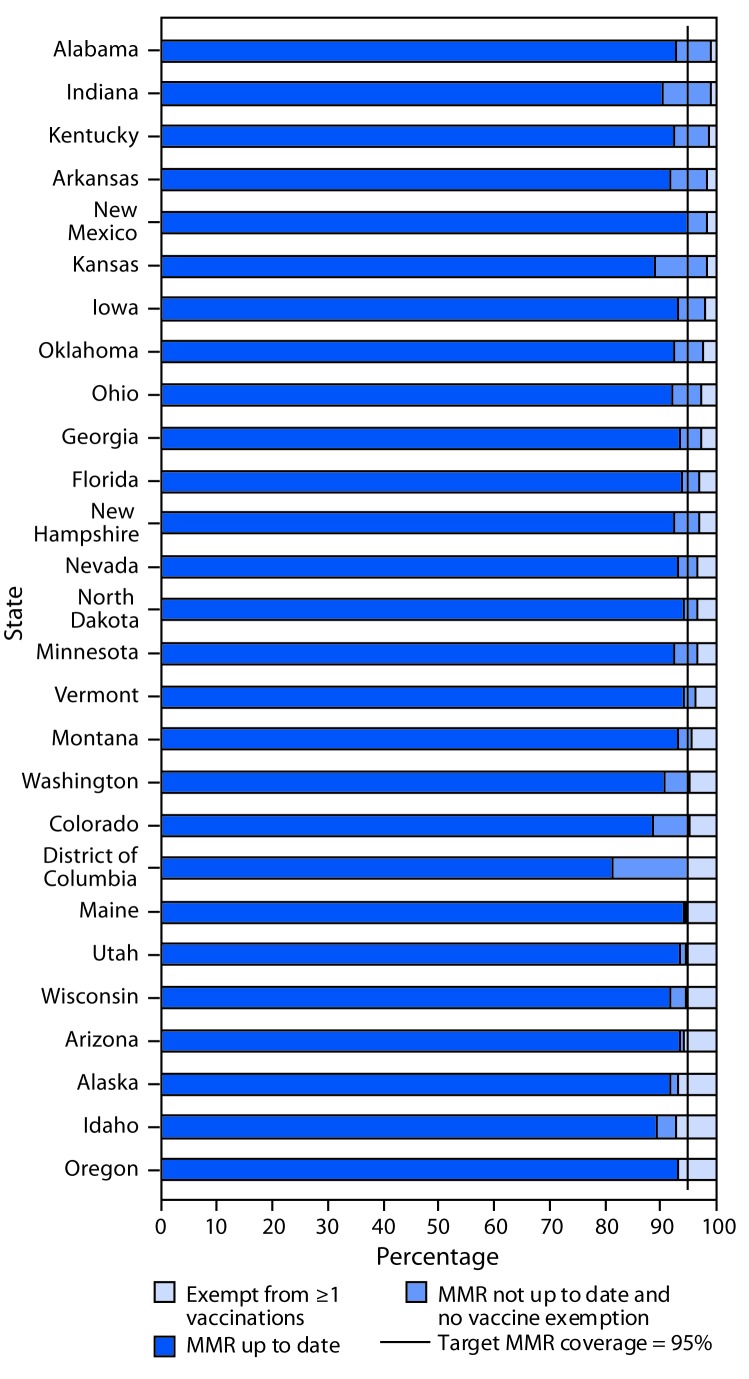
Estimated percentage of kindergartners with documented up-to-date vaccination for measles, mumps, and rubella vaccine (MMR)*; exempt from one or more vaccines^†,§^; and not up to date with MMR and not exempt^¶^ — selected states and District of Columbia,** 2017–18 school year * Estimates are based on completed vaccine series and are not MMR-specific for Alabama, Florida, Georgia, Iowa, and New Hampshire. Up-to-date coverage reported here is the lower bound of possible MMR coverage. ^†^ Most states report the number of kindergartners with an exemption from one or more vaccines. Estimates reported here might include exemptions from vaccines other than MMR, except in Colorado and Minnesota, where MMR-specific exemptions are reported. ^§^ Coverage estimates are based on a sample of kindergartners, and exemption estimates are based on a census for Alaska, Kansas, and Wisconsin. ^¶^ Includes nonexempt students provisionally enrolled, in a grace period, or otherwise without documentation of complete MMR vaccination. ** Figure includes all states with reported MMR coverage for the 2017–18 school year of <95%, the *Healthy People 2020* target for MMR vaccination coverage among kindergartners. https://www.healthypeople.gov/.

